# *catena*-Poly[copper(II)-di-μ-dicyan­amido-μ-1,3-di-4-pyridylpropane]

**DOI:** 10.1107/S1600536809030050

**Published:** 2009-08-08

**Authors:** Jinfang Zhang

**Affiliations:** a Institute of Science and Technology, Jiangsu University, 301 Xuefu Road, Zhenjiang 212013, People’s Republic of China

## Abstract

In the title compound, [Cu(C_2_N_3_)_2_(C_13_H_14_N_2_)]_*n*_, the Cu^II^ atom, located on an inversion centre, adopts a distorted octa­hedral coordination by six N atoms, two from 1,3-di-4-pyridylpropane and four from dicyanamide ligands, with significantly different Cu—N distances. The metal centres are linked in an unusual triple-bridged mode into chains parallel to [101].

## Related literature

For the architectures and topologies of metal-organic compounds, see: Eddaoudi *et al.* (2001 ▶). For their potential applications, see: Zhang *et al.* (2007 ▶); Banerjee *et al.* (2008 ▶). For compounds constructed in single or double-bridged modes, see: Zhang *et al.* (2008 ▶); Lang *et al.* (2004 ▶).
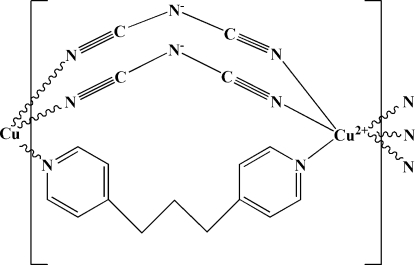


## Experimental

### 

#### Crystal data

[Cu(C_2_N_3_)_2_(C_13_H_14_N_2_)]
*M* *_r_* = 393.91
Monoclinic, 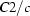

*a* = 16.097 (3) Å
*b* = 10.163 (2) Å
*c* = 12.920 (3) Å
β = 123.10 (3)°
*V* = 1770.6 (6) Å^3^
*Z* = 4
Mo *K*α radiation
μ = 1.25 mm^−1^
*T* = 293 K
0.20 × 0.15 × 0.10 mm

#### Data collection

Rigaku Saturn724+ diffractometer
Absorption correction: multi-scan (*SADABS*; Sheldrick, 1996 ▶) *T*_min_ = 0.798, *T*_max_ = 0.882
4144 measured reflections
1713 independent reflections
1490 reflections with *I* > 2σ(*I*)
*R*_int_ = 0.027

#### Refinement

*R*[*F* ^2^ > 2σ(*F* ^2^)] = 0.059
*wR*(*F* ^2^) = 0.154
*S* = 1.09
1713 reflections
121 parameters
H-atom parameters constrained
Δρ_max_ = 0.42 e Å^−3^
Δρ_min_ = −0.32 e Å^−3^

### 

Data collection: *CrystalClear* (Rigaku, 2008 ▶); cell refinement: *CrystalClear*; data reduction: *CrystalClear*; program(s) used to solve structure: *SHELXS97* (Sheldrick, 2008 ▶); program(s) used to refine structure: *SHELXL97* (Sheldrick, 2008 ▶); molecular graphics: *SHELXTL* (Sheldrick, 2008 ▶); software used to prepare material for publication: *SHELXTL*.

## Supplementary Material

Crystal structure: contains datablocks I, global. DOI: 10.1107/S1600536809030050/pv2188sup1.cif

Structure factors: contains datablocks I. DOI: 10.1107/S1600536809030050/pv2188Isup2.hkl

Additional supplementary materials:  crystallographic information; 3D view; checkCIF report

## Figures and Tables

**Table 1:** Selected bond lengths (Å)

Cu1—N1	2.027 (3)
Cu1—N4	2.031 (4)
Cu1—N2	2.388 (5)

**Fig. 1.**
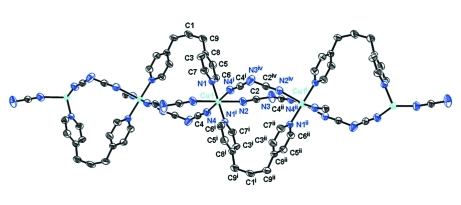
The molecular structure of the title compound, with atomic labels and 30% probability displacement ellipsoids; H atoms have been omitted for clarity.

## supplementary crystallographic information

### Comment

The design and syntheses of metal-organic compounds have attracted great attention in recent years because of not only their intriguing architectures and topologies (Eddaoudi *et al.*, 2001) but also due to their potential applications (Banerjee *et al.*, 2008; Zhang *et al.*, 2007). The flexible bridging ligands can construct metal-organic frameworks with various structures. The tilte compound, (I), was constructed by two kinds of flexible bridging ligands through diffusion reactions of copper(II) nitrate trihydrate, sodium dicyanamide and 1,3-di-4-pyridylpropane which were self-assembled to form a one-dimensional neutral metal-organic compound. In this paper, the crystal structure of (I) is presented.

As illustrated in Fig. 1, Cu^2+^ adopts a distorted octahedral geometry, generated by six nitrogen atoms two from 1,3-di-4-pyridylpropane (bpp) and four from dicyanamide (dca) ligands, Interestingly, the distance Cu1—N2 ([2.388 (5) Å) is significantly longer than those of Cu1—N1 (2.027 (3) Å) and Cu1—N4 (2.031 (4) Å).

Two neighboring Cu atoms are linked by one bpp and two dca ligangs forming a one-dimensional neutral chain in a triple-bridged mode. Compared to single or double-bridged modes (Zhang *et al.*, 2008; Lang *et al.*, 2004), this triple-bridged mode is unfamiliar in coordination compounds.

### Experimental

Cu(NO_3_)_2_.3H_2_O (96.6 mg, 0.4 mmol) was added to 2 ml H_2_O with thorough stirring for 5 minutes and filtered. The blue filtrate was carefully laid on the surface of a solution of bpp (99.1 mg, 0.5 mmol) and NaN(CN)_2_ (89.1 mg, 1 mmol) in 4 ml *i*-PrOH and 2 ml H_2_O. Blue block crystals were obtained after five days.

### Refinement

H atoms were positioned geometrically and refined with riding model, with *U*_iso_ = 1.2*U*_eq_ for methylene and pyridyl H atoms, the C—H bonds are 0.97 Å and 0.93 Å in methylene and pyridyl groups, respectively.

### Crystal data

**Table:**

[Cu(C_2_N_3_)_2_(C_13_H_14_N_2_)]	*F*(000) = 804
*M**_r_* = 393.91	*D*_x_ = 1.478 Mg m^−^^3^
Monoclinic, *C*2/*c*	Mo *K*α radiation, λ = 0.71073 Å
Hall symbol: -C 2yc	Cell parameters from 3458 reflections
*a* = 16.097 (3) Å	θ = 2.6–29.1°
*b* = 10.163 (2) Å	µ = 1.25 mm^−^^1^
*c* = 12.920 (3) Å	*T* = 293 K
β = 123.10 (3)°	Block, blue
*V* = 1770.6 (6) Å^3^	0.20 × 0.15 × 0.10 mm
*Z* = 4	

### Data collection

**Table:**

Rigaku Saturn724+ diffractometer	1713 independent reflections
Radiation source: fine-focus sealed tube	1490 reflections with *I* > 2σ(*I*)
graphite	*R*_int_ = 0.027
dtprofit.ref scans	θ_max_ = 26.0°, θ_min_ = 2.6°
Absorption correction: multi-scan (*SADABS*; Sheldrick, 1996)	*h* = −19→15
*T*_min_ = 0.798, *T*_max_ = 0.882	*k* = −12→12
4144 measured reflections	*l* = −12→15

### Refinement

**Table:**

Refinement on *F*^2^	Secondary atom site location: difference Fourier map
Least-squares matrix: full	Hydrogen site location: inferred from neighbouring sites
*R*[*F*^2^ > 2σ(*F*^2^)] = 0.059	H-atom parameters constrained
*wR*(*F*^2^) = 0.154	*w* = 1/[σ^2^(*F*_o_^2^) + (0.0663*P*)^2^ + 4.1353*P*] where *P* = (*F*_o_^2^ + 2*F*_c_^2^)/3
*S* = 1.09	(Δ/σ)_max_ < 0.001
1713 reflections	Δρ_max_ = 0.42 e Å^−^^3^
121 parameters	Δρ_min_ = −0.32 e Å^−^^3^
0 restraints	Extinction correction: *SHELXTL* (Sheldrick, 2008), Fc^*^=kFc[1+0.001xFc^2^λ^3^/sin(2θ)]^-1/4^
Primary atom site location: structure-invariant direct methods	Extinction coefficient: 0.0025 (8)

### Special details

**Table:**

Experimental. Yield: 82.1 mg in pure form, 52.1% based on Cu. Analysis calculated for C_17_H_14_CuN_8_: C 51.83, H 3.58, N 28.45%; found: C 51.72, H 3.45, N 28.61%. IR: ν, cm^-1^,2182 s, 1606 s, 1424 s, 809 s.
Geometry. All e.s.d.'s (except the e.s.d. in the dihedral angle between two l.s. planes) are estimated using the full covariance matrix. The cell e.s.d.'s are taken into account individually in the estimation of e.s.d.'s in distances, angles and torsion angles; correlations between e.s.d.'s in cell parameters are only used when they are defined by crystal symmetry. An approximate (isotropic) treatment of cell e.s.d.'s is used for estimating e.s.d.'s involving l.s. planes.
Refinement. Refinement of *F*^2^ against ALL reflections. The weighted *R*-factor *wR* and goodness of fit *S* are based on *F*^2^, conventional *R*-factors *R* are based on *F*, with *F* set to zero for negative *F*^2^. The threshold expression of *F*^2^ > σ(*F*^2^) is used only for calculating *R*-factors(gt) *etc*. and is not relevant to the choice of reflections for refinement. *R*-factors based on *F*^2^ are statistically about twice as large as those based on *F*, and *R*- factors based on ALL data will be even larger.

### Fractional atomic coordinates and isotropic or equivalent isotropic displacement parameters (Å^2^)

**Table:**

	*x*	*y*	*z*	*U*_iso_*/*U*_eq_	Occ. (<1)
Cu1	0.2500	0.2500	0.0000	0.0533 (3)	
N1	0.3080 (2)	0.4314 (3)	0.0637 (3)	0.0568 (8)	
N2	0.1117 (3)	0.3051 (5)	0.0129 (5)	0.0969 (16)	
N3	−0.0582 (3)	0.3715 (6)	−0.1106 (4)	0.1082 (19)	
N4	0.3218 (3)	0.1822 (4)	0.1764 (4)	0.0835 (13)	
C1	0.5000	0.8653 (7)	0.2500	0.166 (6)	
H1A	0.5117	0.9240	0.2001	0.199*	0.50
H1B	0.4883	0.9240	0.2999	0.199*	0.50
C2	0.0327 (3)	0.3341 (4)	−0.0500 (4)	0.0614 (11)	
C4	0.3810 (3)	0.1618 (4)	0.2776 (4)	0.0635 (11)	
C5	0.3299 (4)	0.6216 (5)	0.1826 (5)	0.0795 (14)	
H5	0.3203	0.6624	0.2398	0.095*	
C6	0.2971 (3)	0.4963 (5)	0.1450 (4)	0.0680 (12)	
H6	0.2653	0.4539	0.1778	0.082*	
C7	0.3545 (3)	0.4953 (5)	0.0197 (4)	0.0681 (12)	
H7	0.3639	0.4520	−0.0365	0.082*	
C8	0.3770 (3)	0.6876 (5)	0.1364 (5)	0.0771 (15)	
C9	0.4068 (4)	0.8298 (5)	0.1660 (6)	0.101 (2)	
H9A	0.3884	0.8723	0.0891	0.121*	
H9B	0.3655	0.8681	0.1915	0.121*	
C3	0.3891 (4)	0.6210 (5)	0.0527 (5)	0.0757 (13)	
H3	0.4207	0.6615	0.0187	0.091*	

### Atomic displacement parameters (Å^2^)

**Table:**

	*U*^11^	*U*^22^	*U*^33^	*U*^12^	*U*^13^	*U*^23^
Cu1	0.0436 (4)	0.0493 (5)	0.0453 (5)	−0.0036 (3)	0.0103 (3)	−0.0012 (3)
N1	0.0464 (17)	0.0520 (18)	0.0527 (19)	−0.0028 (15)	0.0146 (16)	−0.0036 (16)
N2	0.056 (2)	0.078 (3)	0.097 (3)	0.005 (2)	0.004 (2)	−0.013 (3)
N3	0.068 (3)	0.161 (5)	0.068 (3)	0.028 (3)	0.019 (2)	−0.032 (3)
N4	0.060 (2)	0.071 (3)	0.068 (3)	0.002 (2)	0.002 (2)	−0.004 (2)
C1	0.074 (5)	0.040 (4)	0.226 (12)	0.000	−0.019 (7)	0.000
C2	0.058 (3)	0.058 (2)	0.049 (2)	0.001 (2)	0.017 (2)	−0.001 (2)
C4	0.050 (2)	0.061 (3)	0.060 (3)	−0.004 (2)	0.018 (2)	−0.001 (2)
C5	0.062 (3)	0.071 (3)	0.080 (3)	−0.002 (2)	0.023 (3)	−0.026 (3)
C6	0.061 (2)	0.069 (3)	0.065 (3)	−0.007 (2)	0.028 (2)	−0.013 (2)
C7	0.070 (3)	0.063 (3)	0.064 (3)	−0.006 (2)	0.031 (2)	−0.005 (2)
C8	0.047 (2)	0.049 (3)	0.087 (3)	0.000 (2)	0.006 (2)	−0.005 (3)
C9	0.071 (3)	0.049 (3)	0.120 (5)	0.003 (2)	0.011 (3)	−0.014 (3)
C3	0.068 (3)	0.058 (3)	0.086 (3)	−0.015 (2)	0.033 (3)	0.001 (3)

### Geometric parameters (Å, °)

**Table:**

Cu1—N1^i^	2.027 (3)	C1—H1A	0.9700
Cu1—N1	2.027 (3)	C1—H1B	0.9700
Cu1—N4	2.031 (4)	C4—N3^iv^	1.270 (6)
Cu1—N4^i^	2.031 (4)	C5—C6	1.363 (7)
Cu1—N2	2.388 (5)	C5—C8	1.370 (8)
Cu1—N2^i^	2.388 (5)	C5—H5	0.9300
N1—C7	1.330 (6)	C6—H6	0.9300
N1—C6	1.331 (5)	C7—C3	1.366 (7)
N2—C2	1.112 (6)	C7—H7	0.9300
N3—C4^ii^	1.270 (6)	C8—C3	1.376 (8)
N3—C2	1.283 (6)	C8—C9	1.505 (7)
N4—C4	1.141 (6)	C9—H9A	0.9700
C1—C9^iii^	1.335 (6)	C9—H9B	0.9700
C1—C9	1.335 (6)	C3—H3	0.9300
			
N1^i^—Cu1—N1	180.00 (8)	C9—C1—H1B	99.6
N1^i^—Cu1—N4	90.06 (16)	H1A—C1—H1B	104.1
N1—Cu1—N4	89.94 (16)	N2—C2—N3	172.9 (6)
N1^i^—Cu1—N4^i^	89.94 (16)	N4—C4—N3^iv^	174.1 (6)
N1—Cu1—N4^i^	90.06 (16)	C6—C5—C8	120.0 (5)
N4—Cu1—N4^i^	180.0 (2)	C6—C5—H5	120.0
N1^i^—Cu1—N2	90.08 (16)	C8—C5—H5	120.0
N1—Cu1—N2	89.92 (16)	N1—C6—C5	123.6 (5)
N4—Cu1—N2	88.97 (19)	N1—C6—H6	118.2
N4^i^—Cu1—N2	91.03 (19)	C5—C6—H6	118.2
N1^i^—Cu1—N2^i^	89.92 (16)	N1—C7—C3	123.5 (5)
N1—Cu1—N2^i^	90.08 (16)	N1—C7—H7	118.2
N4—Cu1—N2^i^	91.03 (19)	C3—C7—H7	118.2
N4^i^—Cu1—N2^i^	88.97 (19)	C5—C8—C3	116.9 (4)
N2—Cu1—N2^i^	180.0 (2)	C5—C8—C9	122.3 (5)
C7—N1—C6	116.3 (4)	C3—C8—C9	120.7 (6)
C7—N1—Cu1	120.8 (3)	C1—C9—C8	121.8 (5)
C6—N1—Cu1	122.8 (3)	C1—C9—H9A	106.9
C2—N2—Cu1	138.5 (5)	C8—C9—H9A	106.9
C4^ii^—N3—C2	121.9 (5)	C1—C9—H9B	106.9
C4—N4—Cu1	162.8 (4)	C8—C9—H9B	106.9
C9^iii^—C1—C9	148.6 (8)	H9A—C9—H9B	106.7
C9^iii^—C1—H1A	99.6	C7—C3—C8	119.8 (5)
C9—C1—H1A	99.6	C7—C3—H3	120.1
C9^iii^—C1—H1B	99.6	C8—C3—H3	120.1

Symmetry codes: (i) −*x*+1/2, −*y*+1/2, −*z*; (ii) *x*−1/2, −*y*+1/2, *z*−1/2; (iii) −*x*+1, *y*, −*z*+1/2; (iv) *x*+1/2, −*y*+1/2, *z*+1/2.
